# Cytochrome P450 Herbicide Metabolism as the Main Mechanism of Cross-Resistance to ACCase- and ALS-Inhibitors in *Lolium* spp. Populations From Argentina: A Molecular Approach in Characterization and Detection

**DOI:** 10.3389/fpls.2020.600301

**Published:** 2020-11-16

**Authors:** Marcos Yanniccari, Ramón Gigón, Adelina Larsen

**Affiliations:** ^1^Consejo Nacional de Investigaciones Científicas y Técnicas, Laboratory of Biotechnology and Plant Genetics, Chacra Experimental Integrada Barrow (MDA-INTA), Tres Arroyos, Argentina; ^2^Private Consultant in Weed Control, Tres Arroyos, Argentina; ^3^Instituto Nacional de Tecnología Agropecuaria, Laboratory of Biotechnology and Plant Genetics, Chacra Experimental Integrada Barrow (MDA-INTA), Tres Arroyos, Argentina

**Keywords:** ryegrass, marker-trait associations, multiple-resistance, non-target site resistance (NTSR), target-site resistance (TSR)

## Abstract

Knowledge about the mechanisms of herbicide resistance provide valuable insights into evolving weed populations in response to selection pressure and should be used as a basis for designing management strategies for herbicide-resistant weeds. The selection pressure associated with reactive management against glyphosate-resistant *Lolium* spp. populations would have favored the herbicide resistance to ACCase- and ALS-inhibitors. This work was aimed to determine the sensitivity of 80 Argentinean *Lolium* spp. populations to ALS- and ACCase-inhibitor herbicides for use in wheat or barley and to study the mechanisms of resistance involved. Sensitivity to pinoxaden and iodosulfuron-mesosulfuron were positively correlated (*r* = 0.84), even though both affect different target sites. Inhibitors of cytochrome P450 monooxygenases (P450s) increased the sensitivity to pinoxaden and iodosulfuron-mesosulfuron in 94% of herbicide-resistant populations and target-site ACCase resistance mutations were detected only in two cases. Polymorphic variants were obtained with a pair primer designed on P450 sequences, cluster analysis discriminated around 80% of susceptible and P450-metabolic resistant plants sampled from a single population or different populations. Five markers corresponding to herbicide sensitivity were identified to be significantly associated with phenotypic variance in plants. Resistance to ALS- and ACCase-inhibitor herbicides were closely related, challenging the rotation of herbicides of both sites of action as a practice against resistance. In that sense, the use of pinoxaden and iodosulfuron-mesosulfuron would have provoked a selection on P450 genes that conduced a convergence of P450-metabolism based resistant *Lolium* spp. populations, which was detected by markers in a contribution to elucidate the molecular basis of this type of resistance.

## Introduction

The weeds of farm land are a response to the management applied in the last years (Neve et al., 2009) because farming practices impose selective process on the weed community, conducing shifts in species composition or populations (Darmency, 2019). Thus, herbicide resistance is considered an evolutionary process, where the least herbicide-sensitive plants show an advantage in an environment with herbicide use (Délye et al., 2013). These select plants can involve generalist and/or specialist herbicide adaptations associated to the mechanisms of resistance. Non-target site mechanisms include metabolism or exclusion of the herbicide from the target and they have often been linked to generalist resistance, instead target-site mechanisms have been associated to specialist resistance, including amino acid substitutions, that affect the binding of the herbicide at the target enzyme or overexpression of the target site (Gaines et al., 2020).

Detoxification mechanisms could implicate the most problematic issue for weed management because it often involves unexpected resistance to alternative herbicides or yet undiscovered active principles (Preston, 2004; Yu and Powles, 2014). Herbicide metabolism seems to be controlled by multiple genes encoding enzyme systems, such as cytochrome P450 monooxygenases (P450s) and glutathione S-transferases, responsible for detoxifying non-chemically similar herbicides (Yuan et al., 2007; Yu and Powles, 2014). Specifically, P450s catalyze hydroxylation or dealkylation reactions related to the metabolism of non-related herbicides, such as ACCase- and ALS-inhibitors and glutathione S-transferases, that have been involved in reactions of conjugation to glutathione, directly processing the active herbicide or after the activity of other enzymes, such as P450s (Gaines et al., 2020). P450s conform the largest family of enzymes in plant metabolism, where represent around 1% of the protein-coding genes (Nelson and Werck-Reichhart, 2011). Although the role of P450s in herbicide resistant weeds has been well documented, the genes involved in P450-mediated resistance remained unknown for more than two decades until the detection of two P450 genes (CYP81A12 and CYP81A21) associated with resistance to ALS-inhibitors in *Echinochloa phyllopogon* (Iwakami et al., 2014). Recently, CYP81A genes involved in the detoxification of ACCase-inhibitors have been detected in multiple herbicide resistant *E. phyllopogon* (Iwakami et al., 2019). Moreover, P450 genes and others linked to the metabolism of herbicides have been associated with the increased activity of herbicide detoxification in *Lolium* spp. (Gaines et al., 2014; Duhoux et al., 2017).

Among the most troublesome herbicide-resistant weeds of the world, *Lolium* spp. populations have evolved resistance to at least seven sites of action (Heap, 2020) and new cases of herbicide-resistance are continually detected (Brunton et al., 2019). In Argentina, glyphosate-resistant *Lolium* spp. populations were recorded a decade ago in response to ACCase- and ALS-inhibiting herbicides that have been used to manage these cases at preplant or post-emergence in wheat and barley crops (Yanniccari et al., 2012). However, the selection pressure associated with reactive management against glyphosate-resistant populations would have favored resistance to other principle actives (Yanniccari and Gigón, 2020). In this sense, control failures of *Lolium* spp. with ACCase- and ALS-inhibiting herbicides have been observed frequently in the last few years (Vigna et al., 2017; Gigón and Yanniccari, 2018). This reduces the number of herbicides available for the control of the weed.

*Lolium perenne* ssp. *perenne* and *Lolium perenne* ssp. *multiflorum* or hybrids between them occur in 40% of wheat and barley crops from the Argentinean Pampas (Istilart and Yanniccari, 2012; Scursoni et al., 2014; Yanniccari et al., 2015) and lead to crop yield losses of up to 55% (Scursoni et al., 2012; Gigón et al., 2017). Knowing the status of resistance in *Lolium* spp. populations to ALS and ACCase-inhibiting herbicides used in wheat and barley and the mechanisms of resistance involved could contribute to understanding the evolution of the weed as an input for design strategies of management. The aim of this work was to determine the sensitivity of *Lolium* spp. populations to post-emergence herbicides used in wheat, study the mechanisms of resistance involved and develop candidate P450s based markers associated to herbicide resistance.

## Materials and Methods

### Plant Collection

In December 2015 and 2016, seeds were sampled from 80 *Lolium* spp. populations that had arisen in fallows, wheat, or barley fields in the south of Buenos Aires Province (Supplementary Material). A combination of non-random and random procedures were applied during the selection of sampling points (Beckie et al., 2000; Burgos et al., 2013). The criterion for selecting 17 fields was based on suspicions of herbicide-resistant *Lolium* spp. due to failures of weed control with glyphosate, ACCase-, or ALS-inhibiting herbicides, communicated by technical advisors. Around those sites, four to five *Lolium* spp. populations distanced at least 5 km from each suspicious population were selected at random. One hundred heads from 20 plants were collected at random in each field. Samples were stored under room conditions at 20–25°C for 30 days and later threshed to remove the seeds from the heads, obtaining a bulk sample per population.

### Herbicide-Sensitivity Analysis

In August 2016, 2017, and 2018, seeds of each population were sown in 2-L pots filled with soil to obtain 30 plants per pot. The plants were grown outdoors in a completely randomized design and irrigated at least every 3 days. Iodosulfuron-mesosulfuron (50 and 7.8 g a i L^–1^, respectively, Hussar^®^, Bayer S.A.) and pinoxaden (50 g a i L^–1^ plus cloquintocet-mexyl 12.5 g L^–1^, Axial^®^, Syngenta Agro S.A.) were applied to plants with 2 to 4 expanded leaves at recommended doses (12–1.87 and 40 g a i ha^–1^, respectively) using a precision sprayer calibrated to deliver 200 L ha^–1^. According to manufacturers’ recommendations, 0.2% v/v ethoxylated alcohol was used as a surfactant for iodosulfuron-mesosulfuron spraying. Four untreated pots per population were maintained as controls and four replicates of each herbicide treatment were performed, wherein each pot was a sampling unit.

Plant survival was evaluated after 45 days of herbicide applications, recording the percentage of plants whose growth or color of their leaves was similar to control plants (the number of surviving plants was divided by the total number of plants per pot and multiplied by 100). At the end of the plant life cycle, the production of seeds was checked in pots where surviving plants had previously been registered in order to verify compliance with the definition of herbicide resistance.

A correlation analysis was carried out processing data of plant survival to iodosulfuron-mesosulfuron and pinoxaden with GraphPad Prism^®^ v. 6.01 (GraphPad Software, San Diego, CA, United States). The experiment was replicated twice.

### Analysis of Mechanisms of Resistance

Based on plant survival to iodosulfuron-mesosulfuron or pinoxaden, possible mechanisms of herbicide-resistance were analyzed on populations when ≥40% of plants survived to one or both herbicides:

#### Partial Sequencing of *ACCase* and *ALS* Genes

Total DNA was extracted from five survival plants of each herbicide-resistant population (≥40% of plants survived to one or both herbicides). For that, the CTAB-DTT protocol was carried out following Doyle and Doyle (1990). DNA yield and quality were evaluated spectrophotometrically. The DNA was used as a template to amplify regions of the *ACCase* and *ALS* gene sequences. Two primer pairs (ACCase A (371bp): F 5′-TATGGCTGCAAACTCTGGTG-3′ and R 5′-GTATGCACCGTATGCCAAGT-3′; ACCase B (720bp): F 5′-GGCTCAGCTATGTTCCTGCT-3′ and R 5′-CAAGCCTACCCATGCATTCT-3′) were used according to Matzrafi et al. (2014) and two primer pairs (ALS122-205 (491bp): F 5′-GGGCGCCGACATCCTCGTCG-3′ and R 5′-ATCTGCTG CTGGATGTCCTT-3′; ALS197-574 (1396bp): F 5′-ACTCCAT CCCCATGGTGGC-3′ and R 5′-ATAGGCAGCACATGCTCC TG-3′; ALS574-653 (532bp): F 5′-TGGGCGGCTCAGTA TTACAC-3′ and R 5′-TCCTGCCATCACCTTCCATG-3′) were used following Yu et al. (2008). The PCR amplified fragments were sequenced by Macrogen service (Macrogen Inc., Seoul, South Korea) and the sequence data were cleaned, aligned, translated, and compared at the positions of all known resistance-conferring ACCase and ALS mutations (*ACCase*: Ile-1781, Trp-1999, Trp-2027, Ile-2041, Asp-2078, Cys-2088, and Gly-2096 codons and *ALS*: Ala-122, Pro-197, Ala-205, Asp-376, Arg-377, Trp-574, Ser-653 and Gly-654) using Chromas v.2.6.4 and Bioedit v.7.2. The nucleotide sequences obtained from D-P29 were queried using BLAST of the National Center for Biotechnology Information (NCBI).

#### Detoxification Through Cytochrome P450 Monooxygenases

Herbicide detoxification was evidenced using P450s inhibitors, such as malathion, 1-aminobenzo-triazole (ABT), and piperonyl butoxide (PBO; Matzrafi et al., 2014; Keith et al., 2015). Based on this, the interaction between iodosulfuron-mesosulfuron or pinoxaden and P450s’ inhibitors was evaluated on plumule growth according to Yanniccari and Gigón (2020). A susceptible population (CP-P16) was used as a negative control (Yanniccari et al., 2018). Three grams of seeds of each population were incubated in petri dishes containing a wet filter paper in a growth chamber (75 mmol m^–2^ s^–1^ of photosynthetically active radiation, photoperiod of 12 h, and temperatures of 25 and 20°C for day and night, respectively). After 48 h, germinated seeds with a radicle length of ≥0.2 mm were transferred to 10 mL glass test tubes (four seeds per tube) containing cotton and 1 mL of one of the following treatments: (1.1) deionized water without herbicides or P450 inhibitors (control), (1.2) 10 ppm malathion, (1.3) 10 ppm ABT, (1.4) 20 ppm PBO, (2.1) 1 μM pinoxaden, (2.2) 1 μM pinoxaden + 10 ppm malathion, (2.3) 1 μM pinoxaden + 10 ppm ABT, (2.4) 1 μM pinoxaden + 20 ppm PBO, (3.1) 1 ppm iodosulfuron-mesosulfuron, (3.2) 1 ppm iodosulfuron-mesosulfuron + 10 ppm malathion, (3.3) 1 ppm iodosulfuron-mesosulfuron + 10 ppm ABT, and (3.4) 1 ppm iodosulfuron-mesosulfuron + 20 ppm PBO. Ten tubes were used as replicates for each population and treatment. After incubation for 5 days in a growth chamber under the conditions described above, the plumule length was measured from the point of attachment to the seed to the tip of the coleoptile.

A similar experiment was carried out to determine the response to P450 inhibitors in interaction with different herbicide doses (2, 5, 50, and 100 ppm of iodosulfuron-mesosulfuron or 2, 5, 50, and 100 μM of pinoxaden). This experiment was performed on those resistant populations that did not show response to the inhibitors at 1 ppm of iodosulfuron-mesosulfuron (2.2, 2.3, and 2.4) and 1 μM of pinoxaden (3.2, 3.3, and 3.4).

An ANOVA was performed to evaluate the differences among treatments. When ANOVA indicated significant effects, herbicide treatments with P450 inhibitors were compared to the respective herbicide treatment using Fisher’s protected least significant difference test (*P* < 0.05). The experiment was replicated twice and data from both experiments were pooled when no significant differences between data sets were detected (*P* > 0.05). In all cases, residual plots indicated that variances were normally distributed and homogeneous.

### Molecular Characterization of P450s in Herbicide-Resistant and Susceptible Plants

Susceptible and resistant plants were selected from a unique population (A-P13 with 71 and 63% of plant survival to iodosulfuron-mesosulfuron and pinoxaden respectively, Supplementary Material) in order to have a similar genetic background. Plants were grown for 8 weeks and vegetative clones of individual plants were propagated by tiller partition and repotted to obtain three ramets per plant. When individuals took root, each one was treated with recommended doses of pinoxaden (40 g a i ha^–1^), iodosulfuron-mesosulfuron (12–1.87 g a i ha^–1^), and deionized water as described above. After 45 days, plants were characterized as susceptible or herbicide-resistant (surviving plants to both herbicides). Following the protocol described above, total DNA was isolated from untreated clones of 15 herbicide-resistant plants and 15 susceptible ones. Five primers were designed from consensus regions of 16 P450 sequences obtained by Fischer et al. (2001) and belonging to different cytochrome P450-families. Polymorphic variants on amplified band patterns were obtained with a primer pair (cyp450-F3 5′-TGGGCGATGTCGGTGCTG-3′ and cyp450-hemoR 5′-ACATATTCTAGGTCCCCATCCAAA-3′). The PCR conditions were initial denaturation at 94°C for 2 min and 30 cycles of 94°C for 1 min, 59°C for 1 min, 72°C for 1 min, and final extension at 72°C for 10 min, containing: 300 ηg DNA template, 0.4 μM of each primer, 0.2 mM of each dNTPs, 1.5 mM MgCl_2_, 1X reaction buffer (Inbio Highway), and 1 U Taq polymerase (Inbio Highway) in a 25 μL reaction mix. PCR products were separated by electrophoresis in 1.2% agarose gel stained with ethidium bromide in 0.5× tris-borate-ethylenediaminetetra acetic acid buffer. The results were interpreted using GelAnalyzer^®^ 19.1 and amplified bands were scored as present (1) or absent (0), where bands of an identical molecular weight were considered a similar marker. Genetic similarities were calculated using the Jaccard coefficient and a dendrogram was built from similarities data by unweighted pair group method with arithmetic mean (UPGMA) using the software Infostat^®^ 2017p. GLM association analyses incorporating pairwise kinship information as a covariate were performed employing the R^®^ Core Team 3.6.2 package Genome Association and Prediction Integrated Tool (GAPIT; Lipka et al., 2012). The *P*-values were separately tested using the false positive discovery rate (FDR) test with R^®^ Core Team 3.6.2.

Randomizing the genetic background, a similar experiment was carried out from genomic DNA extracted from 15 herbicide-resistant plants (surviving to pinoxaden or iodosulfuron-mesosulfuron treatments) and 15 susceptible individuals (untreated plants from susceptible accessions) of different populations taken at random within both groups. Data analysis was performed following the methodology detailed above.

## Results

### Herbicide-Sensitivity Analysis

**FIGURE 1:**
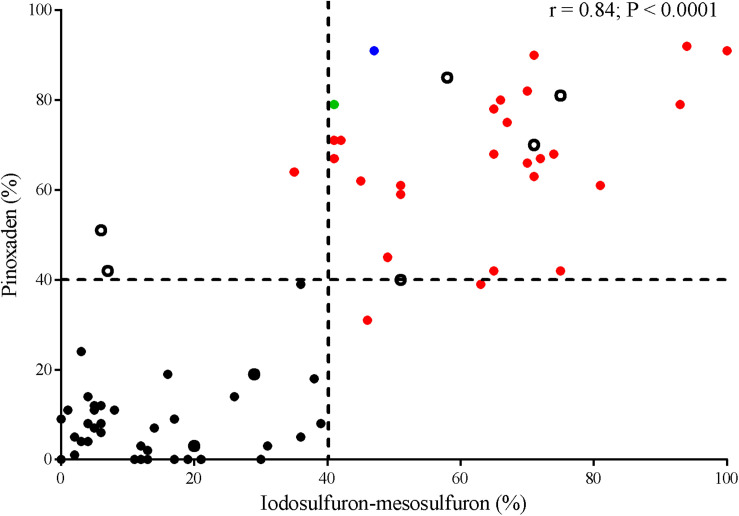
Correlation between plant survival of *Lolium* spp. populations to iodosulfuron-mesosulfuron (ALS-inhibitor) and pinoxaden (ACCase-inhibitor). Discontinued lines indicate a threshold of 40% plant survival to each herbicide. P450-metabolism based resistant (red symbols), TA-P41 (blue symbol), and D-P29 (green symbol) populations. Non-evidence of target-site resistance or herbicide P450-metabolism (open symbols).

Thirty-five *Lolium* spp. populations showed a percentage of pinoxaden- or iodosulfuron-mesosulfuron-resistant plants higher than 40%. An association between sensitivity to both herbicides was detected with a correlation coefficient of 0.84 (Figure 1). Three populations (MH-P33, D-P35, and TA-P41) showed a high proportion (≥75%) of herbicide-resistant plants to one herbicide and a lower percentage of resistance (≤50%) to another principle active (Supplementary Material). In most cases, the differences between plant survival percentages were lower than 30 points (Figure 1).

### Mechanisms of Herbicide-Resistance

**FIGURE 2:**
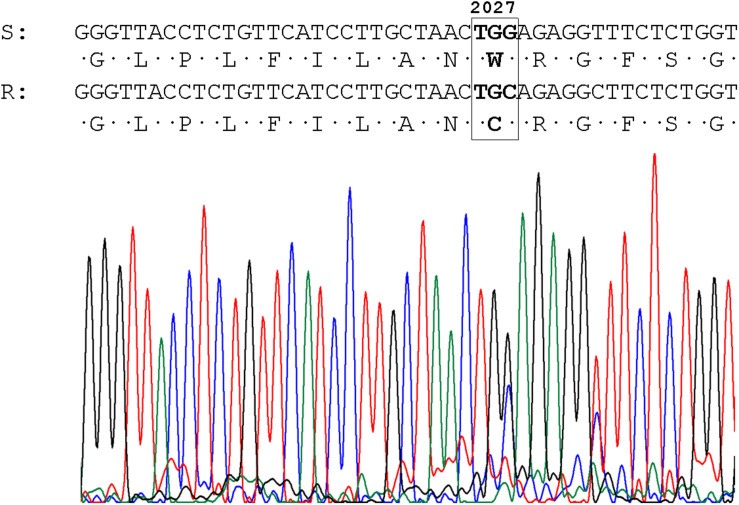
Sequence and chromatogram of the *ACCase* gene obtained from the D-P29 (R) population and the conceptual translation of the amino acid sequence. The resistance-conferring codon is shown in the box. Numbers refer to amino acid positions of full-length ACCase in *Alopecurus myosuroides*.

No evidence of target-site mechanisms of ALS-inhibitor-resistance were found analyzing the codons Ala-122, Pro-197, Ala-205, Asp-376, Arg-377, Trp-574, Ser-653, and Gly-654. However, plants of two populations showed target-site mutations associated with pinoxaden-resistance Asp-2078-Gly in the TA-P41 population (detailed in Yanniccari and Gigón, 2020) and Cys-2027-Trp in D-P29. In the last case, a transversion was detected on the third base of the codon and the mutation was found in a heterozygous state (Figure 2).

**FIGURE 3:**
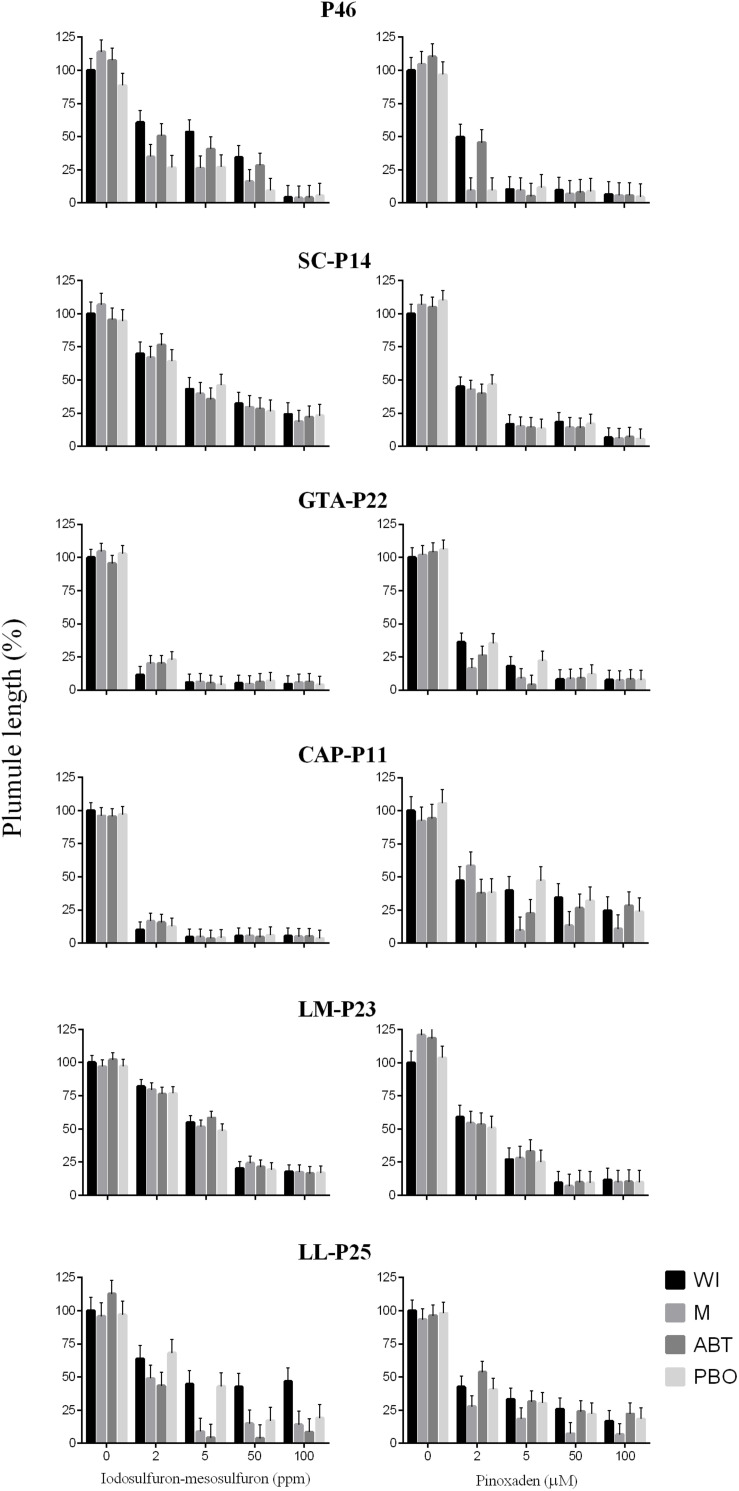
Effects of different doses of iodosulfuron-mesosulfuron and pinoxaden plus malathion (M), 1-aminobenzo-triazole (ABT), piperonyl butoxide (PBO), or without P450-inhibitors (WI) on plumule length of seedlings. Bars represent mean values relatives to the treatment without herbicides or P450-inhibitors (%) and error bars indicate one standard error.

Firstly, the response to P450 inhibitors was detected in 28 populations and depending on the inhibitor used plant responses are rather different among populations (Table 1). Malathion, ABT, or PBO increased the sensitivity to pinoxaden and iodosulfuron-mesosulfuron in 28 cases but in two populations (VE-P0 and D-P29), inhibitors did not affect the response of plumule growth to pinoxaden although it increased the damage provoked by iodosulfuron-mesosulfuron. P450 inhibitors did not condition the response of seven herbicide-resistant populations (P-46, SC-P14, GTA-P22, CAP-P11, TA-P41, LM-P23, and LL-P15) to iodosufuron-mesosulfuron and pinoxaden at a dose of 1 ppm and 1 μM, respectively. 1-Aminobenzo-triazole increased the iodosulfuron-mesosulfuron-sensitivity in 24 cases and only two populations treated with this herbicide showed responses to malathion or PBO without recording a significant effect of ABT. In 26 cases, the action of pinoxaden was exacerbated when it was combined with PBO or ABT (Table 1). Neither P450 inhibitor significantly affected the plumule growth in absence of the herbicide (Supplementary Material). The low pinoxaden-sensitivity of P-46, GTA-P22, and CAP-P11 was reverted with P450 inhibitors at a dose of pinoxaden of >1 μM. In the same way, the response to P450 inhibitors was detected in P-46 and LL-P25 treated with 2–50 ppm and ≥5 ppm of iodosulfuron-mesosulfuron (Figure 3). For two resistant populations (SC-P14 and LM-P23), no evidence of target-site resistance or herbicide P450-metabolism was detected (Figure 3).

**TABLE 1:** Effects of iodosulfuron-mesosulfuron (1 ppm) and pinoxaden (1 μM) plus malathion (M), 1-aminobenzo-triazole (ABT), piperonyl butoxide (PBO), or without P450-inhibitors (WI) on plumule length of seedlings.

**Population**	**Iodosulfuron-mesosulfuron**				**Pinoxaden**			
	**WI**	**M**	**ABT**	**PBO**	**WI**	**M**	**ABT**	**PBO**
A-P13	95	89	52*	55*	44	25*	32	25*
F-P18	89	55*	86	80	35	30	12*	27
DR3	117	70*	65*	58*	70	65	46*	58
LC-P13	97	103	60*	56*	85	58*	81	48*
D-P19	92	88	66*	79	91	97	17*	23*
TA-P24	101	62*	60*	55*	93	59*	66*	57*
E-P32	86	58*	60*	50*	93	53*	47*	41*
MH-P33	90	93	62*	86	72	61	38*	66
D-P35	95	53*	55*	56*	82	42*	48*	47*
V-P39	91	60*	71*	65*	84	40*	49*	43*
P-46	85	83	80	90	70	77	75	67
VE-P0	75	49*	52*	46*	44	37	35	28
SC-P14	86	94	96	92	73	83	78	92
ST-P34	87	77	52*	75	79	73	42*	52*
BW-P12	89	87	51*	65*	48	45	24*	22*
EP-P26	88	80	58*	75	80	53*	42*	38*
CO-P15	103	60	64*	86	85	30*	27*	83
D-C19	88	75	50*	48*	86	96	28*	33*
GTA-P22	31	29	21	20	103	94	90	95
D-P275	93	79	81	44*	47	24*	32	24*
EP-P2	93	63*	61*	52*	87	52*	42*	38*
CAP-P11	36	31	27	38	94	105	109	93
BH-P11	94	105	34*	41*	83	36*	69	45*
TA-P41	49	34	43	37	99	100	99	90
D-P29	64	55	31*	34*	104	95	98	102
L-P4	96	52*	44*	42*	85	49*	50*	41*
LO-P6	92	51*	54*	78	107	36*	51*	82
TP-P14	64	72	58	35*	105	87	52*	59*
LO-P17	70	62	45*	43*	90	54*	45*	41*
LM-P23	98	114	107	111	93	97	103	107
DO-P25	79	57*	72	51*	92	53*	80	48*
LD-P27	86	94	57*	96	96	84	68*	100
DSM-P13	87	97	58*	50*	105	94	61*	55*
D-P15	76	83	53*	51*	90	95	63*	61*
LL-P25	83	79	91	86	88	88	95	85
CP-P16	28	30	26	19	24	19	22	17

Mean values relatives to the treatment without herbicides or P450-inhibitors (%). * Indicate significant effects (*P* ≤ 0.05) of P450-inhibitors compared to the respective control.

### Molecular Characterization of P450s in Herbicide-Resistant and Susceptible Plants

Polymorphic variants were obtained with a pair primer (cyp450-F3 and cyp450-hemoR), where 10 different bands (between 200 and 1950 bp) were detected at least in a frequency of 26% analyzing individuals from a single population (A-P13) or in 13% of plants from different populations.

**FIGURE 4:**
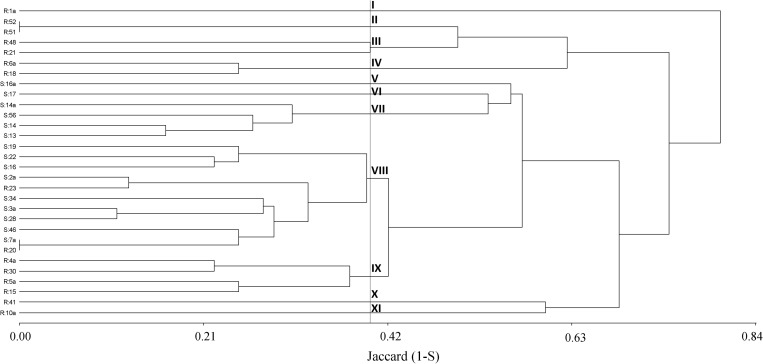
Cluster analysis dendrogram based on ten markers (m1 to m10) detected on 15 herbicide-resistant (R) and 15 susceptible (S) plants from the A-P13 population. Description of clusters I to XII at a cutoff of 50%.

**FIGURE 5:**
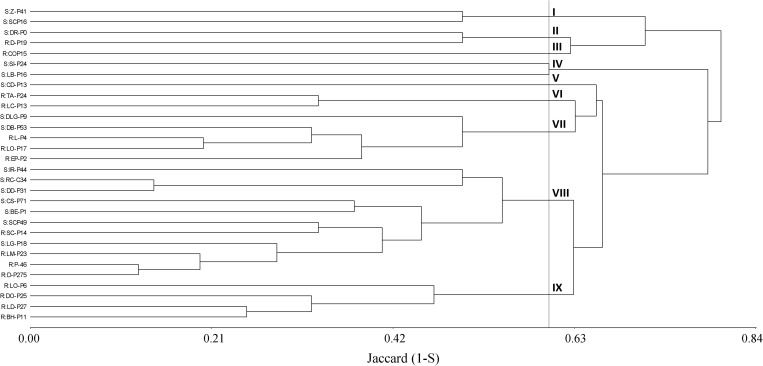
Cluster analysis dendrogram based on ten markers (m1 to m10) detected on 15 herbicide-resistant (R) and 15 susceptible (S) plants from different populations. Description of clusters I to VIII at a cutoff of 60%.

Initially, a dendrogram was constructed on the basis of the similarity index among plants obtained from A-P13. A 50% cutoff value gave 11 distinct clusters where four groups (V, VI, VII, and VIII) were associated with 100% of herbicide-susceptible individuals. In contrast, 7 clusters (I, II, III, IV, IX, X, and XI) contained 86% of pinoxaden and iodosulfuron-mesulfuron-resistant plants (Figure 4). Then, when band patterns were analyzed, considering individuals from thirty populations, 9 clusters were obtained at a cutoff of 60%. Four clusters (I, V, VII, and VIII) grouped 80% of susceptible-individuals, where the biggest group (cluster VIII) included 7 susceptible plants and 4 herbicide-resistant individuals, of which, two were isolated from populations without evidence of P450 metabolism (SC-P14 and LM-P23, Figures 3, 4). Herbicide-resistant individuals were mainly grouped into four clusters (III, VI, VII, and IX), containing 66% of the total resistant plants analyzed (Figure 5).

Both in the intra- and inter-population analysis, three markers were significantly associated with herbicide-sensitivity detected by GLM (Table 2). Of these markers, a band (m7) was linked to the susceptible response to pinoxaden and iodosulfuron-mesosulfuron in both types of analysis and two pair of markers (m2 and m5; m3, and m9) were significantly associated with susceptibility/resistance according to the experiment considered. In all cases, significant effects showed *P*-values ≤0.03 and FDR-adjusted *P*-values ≤0.10 (Table 2).

**TABLE 2:** Marker-trait associations for herbicide-resistance in plants from the A-P13 population and different populations.

**Marker**	**A-P13**		**Populations**	
	**P-value**	**FDR**	**P-value**	**FDR**
m1	1.00	1.00	0.29	0.59
m2	**0.02**	**0.10**	0.72	0.72
m3	0.27	0.39	**0.01**	**0.07**
m4	0.07	0.13	0.16	0.40
m5	**0.03**	**0.10**	0.71	0.72
m6	0.06	0.13	0.71	0.72
m7	**0.01**	**0.10**	**0.01**	**0.07**
m8	0.08	0.13	0.45	0.68
m9	0.46	0.52	**0.02**	**0.07**
m10	0.46	0.52	0.47	0.68

*P-values obtained by GLM analysis and results of false positive discovery rate (FDR) test are shown. *P*-values ≤ 0.05 and FDR-adjusted *P*-values ≤ 0.10 are in bold.*

## Discussion

At a frequency of 40% of resistant plants, a farmer or a technical adviser can detect a failure in chemical control (Burgos et al., 2013) and this threshold was used to compare the herbicide-sensitivity of *Lolium* spp. populations. Notably, the responses of accessions to pinoxaden and iodosulfuron-mesosulfuron were positively correlated, even though both affect different target sites (Figure 1). Thus, populations with a high frequency of pinoxaden-resistant plants (>40%) were also linked to high iodosulfuron-mesosulfuron-resistance and vice-versa. P450-mediated herbicide metabolism seems to be the main mechanism of resistance detected among populations with a high percentage of resistant plants (Figure 1). In these cases, the metabolism of pinoxaden and iodosulfuron-mesosulfuron was reverted by one or more P450-inhibitor, however the three compounds (malathion, ABT, and PBO) were only effective inhibitors of both herbicides in six populations (TA-P24, E-P32, D-P35, V-P39, EP-P2, and L-P4; Table 1). Differential patterns of inhibition of herbicide metabolism by malathion, ABT and PBO could be associated to different metabolic systems, probably involving distinct isoenzymes of P450 (Preston et al., 1996). In four cases (P-46, GTA-P22, CAP-P11, and LL-P25) the P450 inhibitors action as synergists for iodosulfuron-mesosulfuron or pinoxaden was evidenced (Figure 3) at herbicide doses higher than those used to detect the P450-mediated metabolism in most herbicide-resistant populations (Table 1). It will be necessary to determine if an overexpression of P450 or particular isoenzymes are involved in these four populations as found by Iwakami et al. (2014). For SC-P14 and LM-P23 the resistance mechanism(s) are unknown.

P450-metabolism based resistant *Lolium spp.* populations would have evolved resistance under a selection process, tending to favor a generalist herbicide adaptation, despite guaranteeing an adequate rotation of ALS- and ACCase-inhibitor herbicides for use in wheat or barley. It supports the necessity of improving recommendations of herbicide rotations to avoid repeated selection with herbicides that are vulnerable to shared resistance mechanisms (Gaines et al., 2020).

P450 genes would have an important role in the evolution and diversification of organisms, providing adaptive advantages (Omura, 2013). In that sense, markers associated with P450 genes have been highlighted as an efficient tool to study genetic diversity in plants (Ravi et al., 2020). In the current results, cluster analysis discriminated around 80% of susceptible and P450-metabolic resistant plants sampled from a single population or different populations. In the latter case, ten herbicide-resistant populations were mainly grouped into VI, VII, and IX clusters (Figure 5). Interestingly, the three P450 inhibitors increased the iodosulfuron-mesosulfuron or pinoxaden sensitivity in herbicide-resistant populations grouped into VI and VII clusters (TA-P24, LC-P13, L-P4, LO-P17, and EP-P2), however, only one (ABT) or two (malathion and ABT or malathion and PBO) P450 inhibitors affected the herbicide metabolism in D-P275, LO-P6, DO-P25, and LD-P27 grouped into IX cluster (Figure 5). This pattern could support the hypothesis that different P450 isoenzymes are involved in several herbicide-resistant populations. The analyzed markers could be linked to resistance/susceptibility alleles or evidence of the effects of selection pressure on patterns of P450s polymorphism. Nelson and Werck-Reichhart (2011) have pointed out that P450s are an excellent mirror of plant evolution and its role in adaptation.

Beyond the ten markers considered in cluster analysis, through GLM, five markers, corresponding to herbicide sensitivity, including m2, m5, and m7, were identified to be significantly associated with phenotypic variance in plants obtained from the A-P13 population and m3, m7, and m9 explained herbicide sensitivity in individuals from 30 different populations (Table 2). In both experiments, the presence of the m7 band was significantly associated with the herbicide-resistant phenotype. The amplified fragment could be part of one or more resistance genes, or the marker could have no functional role and it could be inherited together with resistance genes. In any case, the results provide the possibility to perform diagnostic prediction of P450s-mediated pinoxaden- and iodosulfuron-mesosulfuron-resistance, the most common mechanism of resistance detected in *Lolium* spp. populations.

Resistance to multiple herbicides emerges as a challenge for current and future weed management. Chemical control practices that seek to reduce selection for specialist resistance traits may promote the evolution of generalist resistance (Comont et al., 2020). The study of metabolic resistance mechanisms and the elucidation of a molecular basis is a difficult and arduous process but a better understanding of these generalist mechanisms will reinforce comprehension of the evolution of weed populations in response to selection pressures and contribute to the development of weed management strategies to delay resistance (Nandula et al., 2019). The findings of the current study indicate that generalist herbicide resistance is due to P450-mediated detoxification, which was highly frequent in *Lolium* spp. populations from Argentinean Pampas. Resistance to ALS- and ACCase-inhibitor herbicides were closely related, challenging the rotation of herbicides of both sites of action as a practice against resistance. Herbicide rotations should be designed to consider the most common mechanisms of resistance associated with each principle active to alternate herbicides commonly conditioned by the same mechanism. In that sense, the use of pinoxaden and iodosulfuron-mesosulfuron would have provoked selection of P450 genes that conduced a convergence of P450-metabolism based resistant in *Lolium* spp. populations, which was detected by markers in a contribution to elucidate the molecular basis of this type of resistance.

## Data Availability Statement

The datasets GENERATED for this study can be found in NCBI GenBank accessions TA-P41: MW178199,D-P29: MW178200.

## Author Contributions

MY and RG conceived, designed, and conducted the experiments. MY wrote the manuscript. All authors analyzed the data, provided editorial advice, and revised manuscript. All authors contributed to the article and approved the submitted version.

## Conflict of Interest

The authors declare that the research was conducted in the absence of any commercial or financial relationships that could be construed as a potential conflict of interest.
